# EarVN1.0: A new large-scale ear images dataset in the wild

**DOI:** 10.1016/j.dib.2019.104630

**Published:** 2019-10-15

**Authors:** Vinh Truong Hoang

**Affiliations:** Faculty of Computer Science, Ho Chi Minh City Open University, Viet Nam

**Keywords:** Ear recognition, Biometric, Image classification, Super-resolution, Image clustering, Right-ear or left-ear detection

## Abstract

Ear recognition is starting to grow as an alternative to other biometric recognition types in recent years. The EarVN1.0 dataset is constructed by collecting ear images of 164 Asian peoples during 2018. It is among the largest ear datasets publicly to the research community which composed by 28,412 colour images of 98 males and 66 females. Thus, this dataset is different from previous works by providing images of both ears per person under unconstrained conditions. The original facial images have been acquired by unconstrained environment including cameras systems and light condition. Ear images are then cropped from facial images over the large variations of pose, scale and illumination. Several machine learning tasks can be applied such as ear recognition, image classification or clustering, gender recognition, right-ear or left-ear detection and enhanced super resolution.

**Table:** Specifications Table

Subject	Computer Vision, Pattern Recognition, Artificial Intelligence
Specific subject area	Ear recognition, Image classification, Biometric identification, Super-resolution, Image clustering
Type of data	Image in RGB colour space
How data were acquired	All images are collected and gathered from volunteer's people from 2018 to 2019 in the unconstrained condition such as illumination, occlusion, rotations and mage resolution.
Data format	.jpeg
Parameters for data collection	Ear images are cropped from daily and portrait photo semi-automatically.
Description of data collection	This dataset consists of 28,412 images of 164 different peoples.
Data source location	Ho Chi Minh City Open University, Ho Chi Minh City, Vietnam
Data accessibility	Mendeley Datahttps://data.mendeley.com/datasets/yws3v3mwx3/3 https://doi.org/10.17632/yws3v3mwx3.3

**Table:** 

**Value of the Data** • This is the largest ear images dataset constructed for biometric recognition. Each subject has at least 100 ear images of the left or right side. • Automatic ear analysis, including tasks such as ear recognition, person identification, image clustering, imbalanced classification might benefit from this dataset. • Some images of this dataset are very low resolution (lower than 25 × 25 pixels) because they are cropped from full facial images. A super-resolution technique could be employed to overcome the inherent resolution limitation. • Gender recognition via ear images can be performed and evaluated on this dataset sine we provide 17,571 ear images of male and 10,841 images of female. • Right-ear or left-ear detection/recognition can be experimented on this dataset. Moreover, an open problem has been raised if a left-ear image can be matched with a right-ear image of the same person.

## 1 Data

Biometric system-based authentication is widely applied for person identification and has many applications such as e-commerce, airport immigration control, device access control, customer authentication, social security, identity check in e-banking … The most successful physiological biometric modalities are fingerprints, irises, and palmprints. In the recent years, ear detection and recognition are an interesting topics of computer vision and widely received attention and research. Ear recognition has more advantages than other biometric features since it has an abundant structure and shape. Moreover, ear has been proved to be a reliable information for discriminating individuals because it is invariant to facial expressions in comparison to the face [4]. Since face of peoples are shaded consciously to attack the surveillance cameras, ear images could be a complement features of biometric system. Various algorithms have been proposed to improve ear recognition performance. For example, Emeršič et al. present a comprehensive review on ear recognition technology by evaluating different features extracted from geometric, holistic, hybrid methods [2] and based on Convolutional Neural Networks (CNN) [9].

We first present a comparison of cropped ear images datasets in Table 1 which illustrate a review of the most popular datasets used for ear recognition research. These databases have different characteristics and variability according to their sources, resolution and number of images per subject. In order to fill the gap and limit of those database, we present a novel ear images dataset namely EarVN1.0. The purpose of this database is to support a large ear images collection for research community.

**Table 1:** Summary of the available ear databases in literature.

Datasets	Country	Number of peoples	Number of images	Image size
IIT Delhi-I [1]	India	121	471	272 × 204
USTB Ear [3]	China	77	308	varied
AWE [2]	Slovenia	100	1000	varied
AWE extend [8]	Slovenia	346	4104	varied
AMI [6]	Spain	106	700	492 × 702
WPUT [5]	Poland	501	2071	varied
UERC [7]	Slovenia	3706	11,804	varied
EarVN1.0	Vietnam	164	28,412	varied and low resolution

**Fig. 1:**
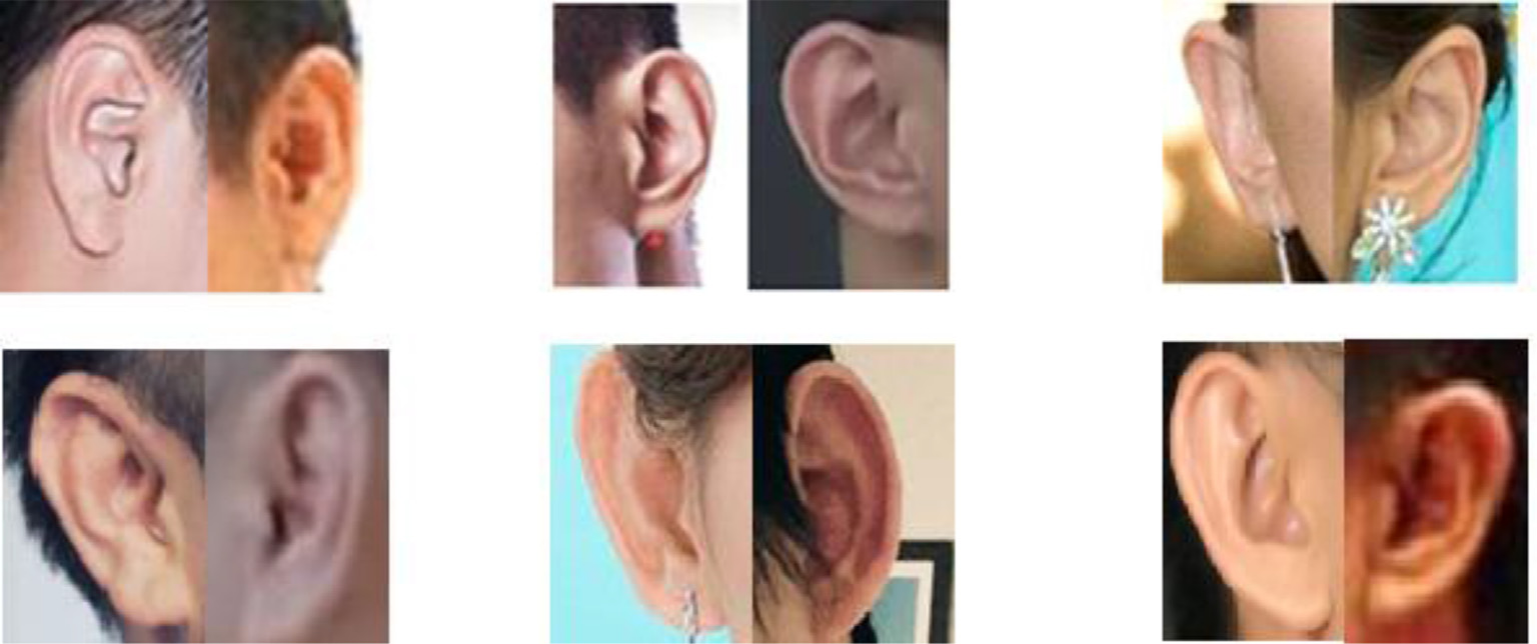
Example of ear images from both sides of six different subjects.

Fig. 1 shows ear images (right and left) of six persons from EearVN1.0 dataset. The two first pair images of the first row are male and the last one is female. All images contain large ear accessories and mixed with other non-interest regions including hair and skin face. Under the current technology condition, these problems make this dataset is more challenging.

## 2 Experimental design, materials, and methods

This data is available online at Mendeley Repertory. It is structured in a main folder (… /images), each subfolder contains all ear images of one person (at least 100 images per person). There are three main tasks can be performed on EarVN1.0 dataset:

- **Ear recognition or person identification via ear:** 164 folders correspond to 164 subjects with 28,412 ear images.
- **Gender recognition/clustering**: there are two classes for male and female recognition/clustering tasks. The first 98 folders (from 01 to 98) is belong to male class and the rest (from 99 to 164) is female.
- **Side-ear detection:** this is the first open problem for ear recognition by identifying the left-ear or right-ear via image. The potential application of this task can be applied for quick authentication. However, the images are not fully labelled, user can apply the semi-supervised learning for this task.
- **Super-resolution:** all ear images of this dataset are on unconstrained low-resolution which has an impact to the performance of biometric systems. We propose to enhance these images to super resolution as an pre-processing step in order to improve the visual clarity and increase the recognition/clustering performance.

## Supplementary material

Data associated with this article can be found in the online version at https://doi.org/10.17632/yws3v3mwx3.3.

## Conflict of Interest

The authors declare that they have no known competing financial interests or personal relationships that could have appeared to influence the work reported in this paper.
